# Predictors of Breastfeeding Initiation Among Postnatal Mothers at Tertiary Care Center of a Tribal Dominant State in India: A Regression Analysis

**DOI:** 10.7759/cureus.16936

**Published:** 2021-08-06

**Authors:** Santosh K Soren, Surendra Sahu, Anit Kujur, Aishwarya Dandpat, Vivek Kashyap, Pragya Kumari, Kumari J Ragini, Archana Kumari, Dewesh Kumar

**Affiliations:** 1 Community Medicine, Rajendra Institute of Medical Sciences, Ranchi, IND; 2 Obstetrics & Gynaecology, Rajendra Institute of Medical Sciences, Ranchi, IND; 3 Obstetrics & Gynaecology, Rajendra Institutue of Medical sciences, Ranchi, Ranchi, IND; 4 Preventive and Social Medicine, Rajendra Institute of Medical Sciences, Ranchi, IND

**Keywords:** breastfeeding, early initiation, postpartum mothers, postnatal ward, regression

## Abstract

Background

Early initiation of breastfeeding (EIBF) is one of the most important predictors for the survival of a child, spacing between two children, and prevention from childhood infections. Breastfeeding plays an important role in reducing child mortality and morbidity but the practice of EIBF globally is way behind the required time for initiation of breastfeeding after delivery. So, we planned to evaluate the early time of initiation of breastfeeding among the postnatal mothers and to determine the predictors of early initiation of breastfeeding in a tertiary hospital setting.

Methodology

A hospital-based cross-sectional study was conducted in the postnatal ward of Rajendra Institute of Medical Sciences (RIMS), Ranchi, Jharkhand for a period of three months (March-May 2017). Our study included 200 postnatal mothers who delivered normal and healthy babies. Mother-infant dyads enrolled in our study were interviewed personally during six hours of the postpartum period. Irrespective of the mode of delivery, all babies born during the study period whose mothers consented to be a part of the study were included. Data collected were entered in MS Excel and analyzed using IBM SPSS Statistics for Windows, Version 20.0. Armonk, NY: IBM Corp.

Results

A total of 200 postnatal mothers fulfilling the study criteria were enrolled during the study. Of them, the majority 98 (49%) belonged to the age group 18-25 years. A hundred and twenty-six (63%) of them resided in rural areas, 182 (91%) were housewives, and only 86 (43%) of them had completed secondary education & above. Early initiation of breastfeeding was found to be only 43 (21.5%) among postnatal mothers. Predictors found to be statistically significant with EIBF were mother's residential status [odds ratio (OR): 2.98; 95% confidence interval (CI): 1.25-7.13], educational status of mother (OR: 3.18; 95% CI: 1.12-9.01) mode of delivery of the baby (OR: 8.93; 95% CI: 2.66-30.06) and ante-natal care (ANC) visits (OR: 0.441; 95% CI: 0.311-0.651). Women’s age, religion, ethnicity, occupation, type of family, and socioeconomic status displayed no statistically significant relationship with EIBF.

Conclusions

It is concluded that nearly one-fifth of mothers in the study initiated breastfeeding within one hour of post-delivery. Maternal education, frequent ANC visits, place of residence, and mode of delivery were also associated with EIBF in India. Nursing staff, as well as clinicians, should reinforce the importance of early initiation of breastfeeding by providing proper health education to post-natal beneficiaries.

## Introduction

Breastfeeding has many health benefits and provides all nutrients to infants in the early months of their life. As per the recommendation of WHO, breastfeeding should be introduced within the first hour after birth [1]. Provision of mother’s milk to infants within an hour of birth is referred to as early initiation of breastfeeding (EIBF). This ensures that the first milk from the mother’s breast also known as colostrum (rich in protective factors) is given to the infants immediately after delivery.

The protective and beneficial effect of EIBF is completely based on the immunological properties present in the mother’s milk. Exclusive breastfeeding during the first six months and avoidance of pre-lacteal foods during the same period ensures proper nutrients and immunoglobulin to babies and helps them to fight against various diseases [2-4]. Although there is evidence supporting the beneficial effect of EIBF, the proportion of EIBF remains low (nearly 50%) in many developing countries including India against the worldwide recommendation of 90% [5-7]. India has made steady progress in raising EIBF rates in the last few decades, but evidence from the regional areas indicate that the percentage of mothers who put their newborn babies on breast milk within the first hour of birth remains abysmally low from the average expected level, which ranges from 36% to 42% [8]. The Lancet report showed that the optimal breastfeeding has a major role in averting 13% of all deaths under the age of two years worldwide [9].

EIBF has many other benefits such as promoting bonding with the mother, maintaining optimal body temperature, and maintaining respiration and blood sugar levels of the newborns. In addition, it is also vital for providing optimal nutrition and development of infants [10]. As per Global Breastfeeding Collective 2017 notes mentioned in WBTi report 2018, despite the exclusive breastfeeding rate being 55%, there have been high mortality in India due to diarrhea and pneumonia in under five children which may be prevented through early initiation of breast feeding, exclusive breast feeding and continued breast feeding up-to 2 years . Inadequate knowledge regarding breastfeeding and frequent use of commercially prepared top feeds were found to be the leading reasons for low breastfeeding rates in the country [11]. Over the years, evidence accumulated reveals the role of multiple socio-cultural, economic, and demographic factors in the promotion of EIBF. Some of these factors which affect EIBF include the gender of the baby, birth order, place of birth, type of delivery and birth, maternal age, maternal education, and religious affiliation. Certain household factors, including the wealth index, rural-urban locality, and region of residence, have also been cited as possible determinants of EIBF [12]. In Jharkhand, there are scanty studies on EIBF, and evidence from tertiary care settings is even less than the community settings. Therefore, the present study was conducted to evaluate the practice of breastfeeding in a tertiary hospital setting, with a focus on the time of initiation of breastfeeding and its determinants among postnatal mothers.

## Materials and methods

The present study was a hospital-based cross-sectional study done at the postnatal ward of the obstetrics department at Rajendra Institute of Medical Sciences (RIMS), Ranchi. Our institute caters to the medical needs of patients coming for medical care irrespective of age, gender, ethnicity, religion, residence, etc. Being a government setup where most of the medical services are free, this hospital is thronged by the population belonging below the poverty line. Approximately 12,000 babies are delivered every year in our hospital. The study was conducted for three months from March 2017 to May 2017 to understand the breastfeeding practices in the hospital settings. Considering the prevalence of initiation of breastfeeding within an hour to be 33% (NFHS 4) [13], power of the study 80%, with 95% confidence interval (CI), precision 7% and 10% additive to total sample size, a total sample size came out to be 198 and rounding it to 200 for our study. A total of 200 mother-baby dyads were randomly selected using the random table method and interviewed in the post-natal ward of the Department of Obstetrics, RIMS, Ranchi. All the pregnant women were enrolled on a structured protocol that contained details of the date of admission, date of discharge, details of vitals, investigation reports, etc. The semi-structured questionnaire which was pretested before the conduction of the study was used for the collection of data which includes parts covering the socio-demographic profile of the mothers and breastfeeding habits. The study was initiated after the prior approval of the Institutional Ethics committee, RIMS, Ranchi. Data was entered in the template created in MS Excel 2017 and all the statistical analysis was done on IBM Corp. Released 2011. IBM SPSS Statistics for Windows, Version 20.0. Armonk, NY: IBM Corp. Frequencies and proportions of the study variables were calculated in our primary analysis for the whole sample. All the factors were cross-tabulated with the prevalence of EIBF to estimate any association. Binary logistic regression was used to evaluate the predictors or drivers associated with EIBF. A p-value of < 0.05 was set to be statistically significant and also their 95% confidence interval was reported in our present study.

## Results

A total of 200 participants were included in our study. The majority of mothers, 98 (49%) were between the age group 18-25 years. Among the newborns, 103 (51.5%) were male and 97 (48.5%) were females. A majority of the mothers were Hindu 122 (61%), 135 (67.5) were of non-tribal ethnicity, 126 (63%) were from rural areas, and 78 (39%) belonged to B.G Prasad class 4 socioeconomic status. About 86 (43%) of the mothers had completed secondary and above education. In our study, most 175 (87.5%) of the babies born were mature, 166 (83%) had normal birth weight, 139 (69.5%) were of either first or second order birth, and 166 (83%) were delivered by caesarian section. According to our study, most of the mothers started breastfeeding within two to four hours but early initiation of breastfeeding was found to be only 43 (21.5%). Among 200 postnatal mothers, the median time of initiation of breastfeeding was three hours, inter-quartile range (IQR) two to five hrs (Table 1).

**Table 1:** Socio-demographic profile of study participants (n=200) # Local Religion of Jharkhand; *As per B.G Prasad Classification

S. No	Characteristics	Category	Frequency n (%)
1	Mother’s Age	18-25 years	98 (49)
		25-30 years	78 (39)
		30-35 years	16 (8)
		35 and above	08 (4)
2	Religion	Hindu	122 (61)
		Muslim	33 (16.5)
		Christian	06 (3)
		Sarna#	35 (17.5)
		Others	04 (2)
3	Ethnicity	Nontribal	135 (67.5)
		Tribal	65 (32.5)
4	Residence	Rural	126 (63)
		Urban	74 (37)
5	Type of Family	Joint	152 (76)
		Nuclear	48 (24)
6	Mother’s Education	Illiterate	53 (26.5)
		Middle school	61 (30.5)
		Secondary and above	86 (43.0)
7	Employment status of mother	Housewife	182 (91)
		Daily wage worker	12 (6)
		Gov. Employee	4 (2)
		Private	2 (1)
8	Socioeconomic status*	Class 1	1 (0.5)
		Class 2	16 (8)
		Class 3	38 (19)
		Class 4	78 (39)
		Class 5	67 (33.5)
9	Total ANC visits	No ANC	47 (23.5)
		1-3ANC	100 (50)
		≥4 ANC	53 (26.5)
10	Gender of newborn	Males	103 (51.5)
		Females	97 (48.5)
11	Birth Order of child	First	69 (34.50
		Second	70 (35.0)
		Third	40 (20.0)
		Fourth	20 (10.0)
		Fifth and above	1 (0.5)
12	Newborn maturity	Mature	175 (87.5)
		Premature	11 (5.5)
		Postmature	14 (7)
13	Birth weight	Normal	166 (83)
		LBW	34 (17)
14	Mode of Delivery	Normal	21 (10.5)
		Assisted	13 (6.5)
		Caesarian	166 (83)
15	Time of initiation of breastfeeding	0 – 2 hrs	70 (350
		2-4 hrs	77 (38.5)
		4-8 hrs	24 (12)
		8 hrs and above	29 (14.5)

Of these 200 women, only 43 (21.5%) reported practicing early initiation of breastfeeding (EIBF). Women of younger age reported a higher proportion of EIBF than women of older age. The educational status of the mother was found to be directly linked with EIBF. Mothers with a higher educational status had a higher proportion of occupational status and were found to have a minor difference in the practice of EIBF with EIBF being slightly more prevalent among working mothers (22.4%) when compared to housewives (21.4%). The third and above birth order children tend to have more chances of early breastfeeding with an EIBF of 28%, compared to the first or second born children (EIBF 18.7%). Female babies (23.7%) reported a higher prevalence of EIBF than male babies (19.4%). Women with babies of small size at birth tend to practice EIBF less than women with standard average or large-sized babies, percentages of EIBF being 9.1%, 21.7%, and 28.6 respectively. Babies who were delivered by normal or assisted delivery had a better EIBF (52.4% and 53.8% respectively) as compared to babies who were delivered by caesarian section (EIBF 15.1%). Mother's residence (rural-urban) was found to have a significant effect on EIBF. Mothers who frequently visited health care centers for antenatal care (ANC) have higher chances of EIBF than mothers with no antenatal visits (Table 2). 

**Table 2:** Association between early initiation of breastfeeding with socio-demographic variables (n=200)

Sr. no	Variables	Category	EIBF N (%)	P value
1	Age of Mothers	18-25yrs	26 (26.5)	
		25-30yrs	13 (16.7)	0.337
		30-35yrs	2 (12.5)	
		≥35yrs	2 (25)	
2	Ethnicity	Nontribal	31 (23)	
		Tribal	12 (18.5)	0.468
3	Awareness of BF	Yes	19 (17.3)	
		No	24 (26.7)	0.121
4	Residence	Urban	08 (10.8)	
		Rural	35 (27.8)	0.005^*^
5	Education	Illiterate	5 (9.4)	
		Middle	16 (26.2)	
		Secondary and above	22 (25.6)	0.044^*^
6	Gender of newborn	Male	20 (19.4)	
		Female	23 (23.7)	0.460
7	Mode of Delivery	Normal	11 (52.4)	
		Assisted	7 (53.8)	
		CS	25 (15.1)	0.000^*^
8	Total ANC visits	No ANC	3 (6.4)	
		1-3 ANC	7 (7)	
		≥4 ANC	33 (62.3)	0.000^*^
9	Birth weight	Normal LBW	37 (22.3) 06 (17.6)	0.548
10	Birth Order	First and Second	26 (18.7)	
		Third and above	17 (27.9)	0.146
11	Maturity of child	Mature	38 (21.7)	
		Premature	1 (9.1)	
		Post mature	4 (28.6)	0.490
12	Family type	Nuclear	11 (22.9)	
		Joint	32 (21.1)	0.784
13	Employment status	Unemployed	39 (21.4)	
		Employed	4 (22.4)	0.938

Multivariate logistic regression analysis was done to find out the various predictors of EIBF. The relationships showing adjusted odds ratios (Adj OR) which are statistically significant with p < 0.05 are described in Table 3. In our study, the women residing in rural areas were three times more likely to have EIBF compared to women residing in urban areas. Children of mothers educated up to middle school were found to have three times more EIBF when compared to the children of illiterate mothers, showing direct association with EIBF and mothers’ education status. Baby delivered by normal vaginal delivery were nine times more likely to have EIBF when compared to a baby delivered by caesarian section. Mothers who did not visit a health center for antenatal check-up were 56% less likely to have EIBF when compared to mothers who frequently visited health centers (Table 3).

**Table 3:** Multivariate logistic regression analysis for determinants of early initiation of breastfeeding

Serial No	Variables	Category	AOR	95% CI	P value
1	Mother’s Age (in years)	18-25 years(Reference)	1		
		25-30 years	1.806	0.857-3.805	0.120
		30-35 years	2.528	0.538-11.885	0.240
		35 yrs and above	1.083	0.206-5.709	0.925
2	Religion	Hindu (Reference)	1		
		Muslim	0.630	0.033-12.045	0.759
		Christian	0.631	0.025-15.698	0.779
		Sarna	1.066	0.382- 2.973	0.999
		Others	0.650	0.038- 11.205	0.767
3	Ethnicity	Tribal	1.097	0.251- 4.786	0.902
		Non tribal (Reference)	1		
4	Education of mother	Illiterate (Reference)	1		
		Middle	3.182	0.123- 9.011	0.029^*^
		Secondary & above	0.952	0.451- 2.011	0.897
5	Employment status of mother	Unemployed (Reference)	1		
		Employed	1.038	0.251- 4.284	0.959
6	Residence	Urban (Reference)	1		
		Rural	2.985	1.249- 7.134	0.014^*^
7	Family type	Joint	0.800	0.289- 2.218	0.668
		Nuclear (Reference)	1		
8	Birth order	First and Second(Reference)	1		
		Third and above	0.516	0.182-1.460	0.212
9	Sex of newborn	Male	1.479	0.630-3.473	0.369
		Female (Reference)	1		
10	Maturity	Mature (Reference)	1		
		Premature	0.296	0.052- 1.690	0.171
		Postmature	4.566	0.614- 33.947	0.138
11	Birth weight	Normal (Reference)	1		
		Low birth weight	1.316	0.381- 4.546	0.664
12	Delivery mode	Normal	8.936	2.656- 30.063	0.000^*^
		Assisted	0.809	0.155- 4.232	0.802
		Caesarean (Reference)	1		
13	Awareness of breastfeeding	Yes	1.742	0.882- 3.438	0.110
		No (Reference)	1		
14	Total ANC visits	No ANC	0.441	0.311-0.651	0.000^*^
		1-3 ANC	0.906	0.224-3.671	0.890
		≥4 ANC (Reference)	1		

## Discussion

Our study done in the Indian tertiary care setup showed that 21.5% of Indian post-natal mothers initiated breastfeeding within an hour of delivery. The proportion and chances of EIBF were found to be highest among Indian mothers who had regular health services visits and those with higher levels of educational status, with major differences in populations residing in rural areas in comparison to the urban population.

Early breastfeeding rates in the state of Jharkhand and the national averages are 32.7% and 50.2%, respectively [13]. Data from the Postgraduate Institute of Medical Education and Research (PGIMER), Chandigarh, suggest that the percentage of EIBF was found to be 64% and cesarean sections attributed to the late initiation of breastfeeding [14]. Report from the tertiary care center of Gujarat reported 32.4%, with maternal fatigue and cesarean section are common predictors for late initiation of breastfeeding [15]. However, in the present study, rates were much lower and the common reasons associated were lower cesarean section and urban population.

Our study indicated that the better academic background of mothers in the total population was associated with EIBF compared to those with poor educational attainment, which was in agreement with the findings from a systematic review conducted for South Asia [16]. Evidence has shown that higher maternal educational levels had a better impact on childhood nutrition and well-being also [17,18]. This may be due to the increased receptivity of a mother with formal education to health promotion campaigns and their empowerment status within the household to make informed health-related decisions [19]. The association between higher maternal educational attainment and EIBF highlights the wide-ranging importance of improving women’s access to quality education, in line with the sustainable development goal-4 (SDG-4), which aims to ensure that all girls and boys complete free, equitable, and quality primary and secondary education by the year 2030 [20].

Difficulties in accessing health care services such as ANC have been found to be significant barriers in initiating breastfeeding within the first hour of birth [21]. Our study indicated that receiving four or more ANC sessions was associated with an increased likelihood of EIBF in the total population. This finding is congruous with previous studies conducted in Sri Lanka, and Bangladesh which showed that no or less than four ANC visits were associated with delayed initiation of breastfeeding [22,23]. These findings suggest that the health messages provided during ANC sessions could improve mothers’ adherence to the WHO breastfeeding recommendations [24]. For example, step 3 of the revised baby-friendly hospital initiative (BFHI) indicates that all pregnant women and their families should be informed of the importance and management of breastfeeding in ANC sessions [25].

This study shows differences in rural and urban areas in relation to EIBF. Better maternal educational background and frequent ANC (≥ 4) visits were associated with EIBF. Mothers in rural areas are more inclined to timely initiate breastfeeding; this is mainly due to government-funded rural specific maternal and child health (MCH) interventions which aimed to address the health needs of under-served rural areas [26].

Furthermore, the mode of delivery also has a great role in determining EIBF, with cesarean sections potentially negating some of the positive effects of vaginal delivery. Consistent with previous Indian studies, as well as the broader literature, we found that cesarean section was associated with delayed EIBF compared to vaginal birth [5]. A recent systematic review suggests that the negative impact of cesarean section on EIBF may be due to the post-operative care that hampers the early skin-to-skin contact of mother and baby which supports EIBF [27]. Despite this challenge, evidence suggests that EIBF is feasible even after cesarean delivery if health professionals are well-trained to provide the necessary support and guidance to the mother [28]. It is, therefore, essential that initiatives aimed at increasing breastfeeding should include the training of health professionals and the establishment of ‘baby friendly’ health facilities to appropriately support mothers to breastfeed within the first hour of birth [29].

The study has methodological limitations that should be considered. We have conducted a cross-sectional study for data collection, so a clear temporal association between the study factors and EIBF cannot be established.

## Conclusions

Our study concluded that nearly one-fifth of mothers in the study initiated breastfeeding within one hour of post-delivery. Maternal education, frequent ANC visits, place of residence, and mode of delivery were also associated with EIBF in India. The abysmally low prevalence of EIBF in tertiary care settings is a major concern and needs to be addressed at the hospital level as it shows a major gap in practices and a lack of health education. The health personnel should be motivated to counsel about the importance of early initiation of breastfeeding to all mothers with special attention to the predictors highlighted in the study. Continuous re-enforcement of the importance of EIBF by senior consultants and obstetricians to nursing staff will also help in increasing the prevalence of EIBF. The steps of the BFHI should be internally assessed and monitored to reap its benefits. More evidence is needed from major tertiary care settings in this regard to get a more clear picture of the present situation.

## References

[1] (2021). World Health Organisation. Breastfeeding. Available. http://from:https://www.who.int/maternal_child_adolescent/topics/newborn/nutrition/breastfeeding/en/.

[2] Wang H, Bhutta AZ, Coates MM (2016). Global, regional, national, and selected subnational levels of stillbirths, neonatal, infant, and under-5 mortality, 1980-2015: a systematic analysis for the Global Burden of Disease Study 2015. Lancet.

[3] Ogbo FA, Agho K, Ogeleka P, Woolfenden S, Page A, Eastwood J (2017). Infant feeding practices and diarrhoea in sub-Saharan African countries with high diarrhoea mortality. PLoS One.

[4] Khan J, Vesel L, Bahl R, Martines JC (2015). Timing of breastfeeding initiation and exclusivity of breastfeeding during the first month of life: effects on neonatal mortality and morbidity--a systematic review and meta-analysis. Matern Child Health J.

[5] Takahashi K, Ganchimeg T, Ota E (2017). Prevalence of early initiation of breastfeeding and determinants of delayed initiation of breastfeeding: secondary analysis of the WHO global survey. Sci Rep.

[6] Jones G, Steketee RW, Black RE, Bhutta ZA, Morris SS (2003). How many child deaths can we prevent this year?. Lancet.

[7] Aguayo VM, Gupta G, Singh G, Kumar R (2016). Early initiation of breast feeding on the rise in India. BMJ Glob Health.

[8] Badaya N, Jain S, Kumar N (2018). Time of initiation of breastfeeding in various modes of delivery and to observe the effect of low birth weight and period of gestation on initiation of breastfeeding. Int J Contemp Pediatr.

[9] (2021). UNICEF. https://www.unicef.org.uk/babyfriendly/lancet-increasing-breastfeeding-worldwide-prevent-800000-child-deaths-every-year/.

[10] (2021). Alive and thrive. Centers of excellence for breastfeeding. https://www.aliveandthrive.org/en/how-we-work/centers-of-excellence-for-breastfeeding.

[11] (2020). World Breastfeeding Trends Initiative (WBTi). Arrested development. 5th Report of India's policy and programmes on infant and young child feeding. India Report.

[12] Islam MA, Mamun A, Hossain MM, Bharati P, Saw A, Lestrel PE, Hossain MG (2019). Prevalence and factors associated with early initiation of breastfeeding among Bangladeshi mothers: a nationwide cross-sectional study. PLoS One.

[13] NFHS NFHS (2020). National Family Health Survey Report 2015‑2016. http://rchiips.org/nfhs/NFHS- 4Reports/India.pdf.

[14] Raghavan V, Bharti B, Kumar P, Mukhopadhyay K, Dhaliwal L (2014). First hour initiation of breastfeeding and exclusive breastfeeding at six weeks: prevalence and predictors in a tertiary care setting. Indian J Pediatr.

[15] Bhatt S, Parikh P, Kantharia N, Dahat A, Parmar R (2012). Knowledge, attitudeand practice of postnatal mothers for early initiation of breast feedingin the obstetric wards of a tertiary care hospital of Vadodara city. Natl J community Med.

[16] Sharma IK, Byrne A (2016). Early initiation of breastfeeding: a systematic literature review of factors and barriers in South Asia. Int Breastfeed J.

[17] Afnan-Holmes H, Magoma M, John T (2015). Tanzania's countdown to 2015, an analysis of two decades of progressand gaps for reproductive, maternal, newborn, and child health, to informpriorities for post-2015. Lancet Glob Health.

[18] Ogbo FA, Dhami MV, Awosemo AO (2019). Regional prevalence and determinants of exclusive breastfeeding in India. Int Breastfeed J.

[19] Acharya P, Khanal V (2015). The effect of mother's educational status on early initiation of breastfeeding: further analysis of three consecutive Nepal demographic and health Surveys. BMC Public Health.

[20] (2021). Sustainable development goals: take action for the sustainable development goals. https://www.un.org/sustainabledevelopment/sustainable-development-goals/.

[21] Adam T, Lim SS, Mehta S (2005). Cost effectiveness analysis of strategies for maternal and neonatal health in developing countries. BMJ.

[22] Senarath U, Dibley MJ, Godakandage SS, Jayawickrama H, Wickramasinghe A, Agho KE (2010). Determinants of infant and young child feeding practices in Sri Lanka: secondary data analysis of demographic and health survey 2000. Food Nutr Bull.

[23] Mihrshahi S, Kabir I, Roy SK, Agho KE, Senarath U, Dibley MJ (2010). Determinants of infant and young child feeding practices in Bangladesh: secondary data analysis of demographic and health survey 2004. Food Nutr Bull.

[24] Renkert S, Nutbeam D (2001). Opportunities to improve maternal health literacy through antenatal education: an exploratory study. Health Promot Int.

[25] (2021). Implementation guidance: protecting, promoting and supporting breastfeeding in facilities providing maternity and newborn services - the revised baby-friendly hospital initiative. GenevaWorld Health Organization.

[26] Gupta M, Angeli F, van Schayck OC, Bosma H (2015). Effectiveness of a multiple-strategy community intervention to reduce maternal and child health inequalities in Haryana, North India: a mixed-methods study protocol. Glob Health Action.

[27] Prior E, Santhakumaran S, Gale C, Philipps LH, Modi N, Hyde MJ (2012). Breastfeeding after cesarean delivery: a systematic review and meta-analysis of world literature. Am J Clin Nutr.

[28] Ogbo FA, Eastwood J, Page A (2016). Prevalence and determinants of cessation of exclusive breastfeeding in the early postnatal period in Sydney, Australia. Int Breastfeed J.

[29] Desai G, Anand A, Modi D (2017). Rates, indications, and outcomes of caesarean section deliveries: a comparison of tribal and non-tribal women in Gujarat, India. PLoS One.

